# Open vs totally laparoscopic right colectomy: technique and results

**DOI:** 10.1186/1471-2482-13-S1-A20

**Published:** 2013-09-16

**Authors:** Massimiliano Fabozzi, Rosaldo Allieta, Luciano Grimaldi, Stefano Reggio, Bruno Amato, Michele Danzi

**Affiliations:** 1 Department of General Surgery, “U. Parini” Hospital, Aosta, Italy; 2 Department of Specialized Surgery, Division of Gastrointestinal Surgery Rehabilitation of Election and Emergency. “Federico II” University, Naples, Italy

## Background

Right colectomy is the surgical treatment for malignant pathologies involving the intestinal tract between the ileocecal Bahuino valve and the colic hepatic flexure. Laparoscopic resection must respect the same oncological criteria as the open approach including: ''no-touch isolation technique'', isolation and ligation of the vascular pedicles at the origin, oncological lymphadenectomy and ''distal and radial clearance'' of the neoplasm from resection margins.

**Figure 1:**
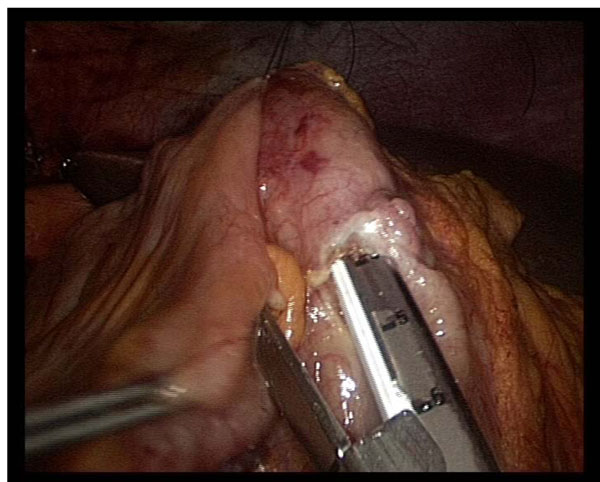
**Intraoperative image** Laparoscopic ileo-colic anastomosis.

Two major procedures have been described for the treatment of right colon tumors: Open right colectomy (ORC) and Totally Laparoscopic resection (TL) in which vascular ligations, intestinal resection and anastomosis are performed by laparoscopy (Figure 1).

In ORC technique, there is an abdominal right side laparotomy; in TL there is a minilaparotomy used only for endobag colon extraction and it is located in parapubic region.

## Methods

From May 2004 to march 2013, we performed in High Specialistic Surgical Centers (Aosta "Parini" Hospital and Naples "Federico II" University) 132 laparoscopic right colectomies and 127 open right colectomies of which we have selected 75 laparoscopic cases of these 11 for benign pathologies and 64 for neoplastic diseases and 75 Open Cases. The M/F rate was 1/1. The mean age was 64.7 ± 7.2.

Colonic preoperative washout was performed to all patients with 2 L for a day of polyethylene glycol (PEG) in the two days before the operation, associated with a fiber-free diet. The day before operation, we positioned in all patient antalgic peridural catheter with 0.5% levobupivacaine (4 ml/h); on the following day, in the operating room, after anesthetic induction, we also positioned nasogastric tube (NG tube) and urinary catheter (UC) and no drain according to Kehlet protocols (in the last 23 cases). In the TL colectomy, the sovrapubic minilaparotomy of 6 ± 2 cm is necessary only for the specimen extraction from the parapubic minilaparotomy performed by a 15-mm Endocatch, preventing the peritoneal spreading of neoplastic cells.

The procedures were considered curative only when there was no intraoperative evidence of secondary locations.

NG tube was removed after the operation and UC in the morning after surgery. The patients were allowed to drink liquids with oral assumption of medicines the evening of the operation (Table 1) [1]. All the patients underwent a cycle of postoperative physiokinesis therapy. Patients were discharged when they became autonomous in movements and walking with a restored bowel function without fever and pain.

**Table 1:** Patients data and operative parameters

	Open right colectomy	Totally Laparoscopic
**Patients number**	75	75
**Age**	62.4±7.5 years	64.7±7.1 years
**Sex**	43 F / 32 M	40 / 35M
**BMI (kg/m^2^)**	25.1±1.5	26.2±2.7
**ASA I**	32	28
**ASA II**	38	43
**ASA III**	5	4
**NG tube removal**	1st p.o. day	evening of surgery
**Water assumption**	2nd p.o. day	evening of surgery
**Urinary catheter**	1st p.o. day	1st p.o. day
**Time of refeeding**	3rd p.o. day	1st p.o. day

They were followed-up at least 1 year, starting on the 30^th^ postoperative day and then at 3, 6 and 12 months from the operation. After the first year the patients were followed-up each 6 months until the 5^th^ p.o. year.

## Results

The results are shown in Table 2: the mean operative time was similar between the two groups whereas the data related to p.o. pain, analgesic consumption and digestive function restoration was better in TL group compared to ORC group. The mean hospital stay was about 5 days in TL vs 7 days in ORC tecnique. There were no post-operative complications and there was no mortality in the TL group. There wasn’t recurrence of the neoplastic disease in both groups after five years of follow-up [4].

**Table 2:** Outcomes

Outcomes	Open right colectomy	Totally laparoscopic
**Operative time** (min.)	74±27	76±22
**Intra-operative complications** (small bowel lesion)	0	1(1.33%)
**Laparotomy size** (cm)	16±1	6±2
**First peristalsis** (days)	2.7±0.3	1.2±0.8
**First defecation** (days)	3.4±0.7	3.2±1.1
**Permanence of drain** (days)	2.3±1.6	1.1±1.3 (No drain in the last 23 cases)
**Post-operative pain** (VAS pain scale)	6.6	2.1
**Analgesic duration therapy**(days)	5. 8±1.6	2.1±1.2
**Post-operative complications** (number, rate)	9	0
**Hospital stay** (days)	7.6±1.2	5.1±1.3
**Tumor recurrence**	0	0
**Mortality**	0	0

## Conclusions

TL approach seems to present some advantages [2,3]: 1. **Functional**: The smaller size of Parapubic minilaparotomy, with a low risk of infection and incisional hernias, reduced postoperative pain, determined an inferior risk of respiratory infection and a better postoperative course and subsequently a shorter hospitalization and reduced assistance costs. The laparotomy of "open" right colectomies instead, is of larger size, with higher risk of wound infection; the upper site of right abdominal incision determined higher p.o. pain with reduced diaphragmatic respiratory excursions and higher risk of respiratory infection expecially in elder. 2. **Technical**: the facilitated closure of the mesos by laparoscopy in TL right colectomy avoids internal hernias, and the absence of mesos traction during the laparoscopic anastomosis allows a faster restoration of peristalsis; 3. **Anesthetic:** thanks to the smaller size of parapubic minilaparotomy. In conclusion according to results of this study TL right colectomy seems to be a feasible and safe tecnique with the same oncological results of the open approach but with an improved post-operative patient’s comfort, however it is necessary to conduct further perspective studies to draw definitive conclusion.
